# Supplementary material for: A fault-intrusion-tolerant system and deadline-aware algorithm for scheduling scientific workflow in the cloud
Source: PeerJ Comput Sci. 2021 Nov 2;7:e747. doi: 10.7717/peerj-cs.747 (PMC8576554; doi:10.7717/peerj-cs.747)
Supplement: Supplemental Information 6 [file peerj-cs-07-747-s006.zip › Myproject1.1/docs/org/cloudbus/cloudsim/power/PowerVm.html]

PowerVm


JavaScript is disabled on your browser.


- Overview
- Package
- Class
- Use
- Tree
- Deprecated
- Index
- Help

- Prev Class
- Next Class

- Frames
- No Frames

- All Classes

- Summary:
- Nested |
- Field |
- Constr |
- Method

- Detail:
- Field |
- Constr |
- Method


org.cloudbus.cloudsim.power

## Class PowerVm

- java.lang.Object
- - org.cloudbus.cloudsim.Vm
  - - org.cloudbus.cloudsim.power.PowerVm

- ---

    

  ```
  public class PowerVm
  extends Vm
  ```

  The class of a VM that stores its CPU utilization history. The history is used by VM allocation
  and selection policies.
  If you are using any algorithms, policies or workload included in the power package please cite
  the following paper:
  Anton Beloglazov, and Rajkumar Buyya, "Optimal Online Deterministic Algorithms and Adaptive
  Heuristics for Energy and Performance Efficient Dynamic Consolidation of Virtual Machines in
  Cloud Data Centers", Concurrency and Computation: Practice and Experience (CCPE), Volume 24,
  Issue 13, Pages: 1397-1420, John Wiley & Sons, Ltd, New York, USA, 2012

  Since:
  :   CloudSim Toolkit 2.0

- - ### Field Summary

    Fields

    | Modifier and Type | Field and Description |
    | --- | --- |
    | `static int` | `HISTORY_LENGTH` The Constant HISTORY\_LENGTH. |
  - ### Constructor Summary

    Constructors

    Constructor and Description

    `PowerVm(int id,
    int userId,
    double mips,
    int pesNumber,
    int ram,
    long bw,
    long size,
    int priority,
    java.lang.String vmm,
    CloudletScheduler cloudletScheduler,
    double schedulingInterval)`

    Instantiates a new power vm.
  - ### Method Summary

    Methods

    | Modifier and Type | Method and Description |
    | --- | --- |
    | `void` | `addUtilizationHistoryValue(double utilization)` Adds the utilization history value. |
    | `double` | `getPreviousTime()` Gets the previous time. |
    | `double` | `getSchedulingInterval()` Gets the scheduling interval. |
    | `protected java.util.List<java.lang.Double>` | `getUtilizationHistory()` Gets the utilization history. |
    | `double` | `getUtilizationMad()` Gets the utilization MAD in MIPS. |
    | `double` | `getUtilizationMean()` Gets the utilization mean in percents. |
    | `double` | `getUtilizationVariance()` Gets the utilization variance in MIPS. |
    | `void` | `setPreviousTime(double previousTime)` Sets the previous time. |
    | `protected void` | `setSchedulingInterval(double schedulingInterval)` Sets the scheduling interval. |
    | `double` | `updateVmProcessing(double currentTime, java.util.List<java.lang.Double> mipsShare)` Updates the processing of cloudlets running on this VM. |

    - ### Methods inherited from class org.cloudbus.cloudsim.Vm

      `addStateHistoryEntry, getBw, getCloudletScheduler, getCurrentAllocatedBw, getCurrentAllocatedMips, getCurrentAllocatedRam, getCurrentAllocatedSize, getCurrentRequestedBw, getCurrentRequestedMaxMips, getCurrentRequestedMips, getCurrentRequestedRam, getCurrentRequestedTotalMips, getHost, getId, getMips, getNumberOfPes, getRam, getSize, getStateHistory, getTotalUtilizationOfCpu, getTotalUtilizationOfCpuMips, getUid, getUid, getUserId, getVmm, isBeingInstantiated, isInMigration, setBeingInstantiated, setBw, setCloudletScheduler, setCurrentAllocatedBw, setCurrentAllocatedMips, setCurrentAllocatedRam, setCurrentAllocatedSize, setHost, setId, setInMigration, setMips, setNumberOfPes, setRam, setSize, setUid, setUserId, setVmm`
    - ### Methods inherited from class java.lang.Object

      `clone, equals, finalize, getClass, hashCode, notify, notifyAll, toString, wait, wait, wait`

- - ### Field Detail


    - #### HISTORY\_LENGTH

      ```
      public static final int HISTORY_LENGTH
      ```

      The Constant HISTORY\_LENGTH.

      See Also:
      :   Constant Field Values
  - ### Constructor Detail


    - #### PowerVm

      ```
      public PowerVm(int id,
             int userId,
             double mips,
             int pesNumber,
             int ram,
             long bw,
             long size,
             int priority,
             java.lang.String vmm,
             CloudletScheduler cloudletScheduler,
             double schedulingInterval)
      ```

      Instantiates a new power vm.

      Parameters:
      :   `id` - the id
      :   `userId` - the user id
      :   `mips` - the mips
      :   `pesNumber` - the pes number
      :   `ram` - the ram
      :   `bw` - the bw
      :   `size` - the size
      :   `priority` - the priority
      :   `vmm` - the vmm
      :   `cloudletScheduler` - the cloudlet scheduler
      :   `schedulingInterval` - the scheduling interval
  - ### Method Detail


    - #### updateVmProcessing

      ```
      public double updateVmProcessing(double currentTime,
                              java.util.List<java.lang.Double> mipsShare)
      ```

      Updates the processing of cloudlets running on this VM.

      **Overrides:**
      :   `updateVmProcessing` in class `Vm`

      Parameters:
      :   `currentTime` - current simulation time
      :   `mipsShare` - array with MIPS share of each Pe available to the scheduler

      Returns:
      :   time predicted completion time of the earliest finishing cloudlet, or 0 if there is
          no next events


    - #### getUtilizationMad

      ```
      public double getUtilizationMad()
      ```

      Gets the utilization MAD in MIPS.

      Returns:
      :   the utilization mean in MIPS


    - #### getUtilizationMean

      ```
      public double getUtilizationMean()
      ```

      Gets the utilization mean in percents.

      Returns:
      :   the utilization mean in MIPS


    - #### getUtilizationVariance

      ```
      public double getUtilizationVariance()
      ```

      Gets the utilization variance in MIPS.

      Returns:
      :   the utilization variance in MIPS


    - #### addUtilizationHistoryValue

      ```
      public void addUtilizationHistoryValue(double utilization)
      ```

      Adds the utilization history value.

      Parameters:
      :   `utilization` - the utilization


    - #### getUtilizationHistory

      ```
      protected java.util.List<java.lang.Double> getUtilizationHistory()
      ```

      Gets the utilization history.

      Returns:
      :   the utilization history


    - #### getPreviousTime

      ```
      public double getPreviousTime()
      ```

      Gets the previous time.

      Returns:
      :   the previous time


    - #### setPreviousTime

      ```
      public void setPreviousTime(double previousTime)
      ```

      Sets the previous time.

      Parameters:
      :   `previousTime` - the new previous time


    - #### getSchedulingInterval

      ```
      public double getSchedulingInterval()
      ```

      Gets the scheduling interval.

      Returns:
      :   the schedulingInterval


    - #### setSchedulingInterval

      ```
      protected void setSchedulingInterval(double schedulingInterval)
      ```

      Sets the scheduling interval.

      Parameters:
      :   `schedulingInterval` - the schedulingInterval to set


- Overview
- Package
- Class
- Use
- Tree
- Deprecated
- Index
- Help

- Prev Class
- Next Class

- Frames
- No Frames

- All Classes

- Summary:
- Nested |
- Field |
- Constr |
- Method

- Detail:
- Field |
- Constr |
- Method
